# Health Risk Assessment for Inhalation Exposure to Methyl Tertiary Butyl Ether at Petrol Stations in Southern China

**DOI:** 10.3390/ijerph13020204

**Published:** 2016-02-06

**Authors:** Dalin Hu, Jianping Yang, Yungang Liu, Wenjuan Zhang, Xiaowu Peng, Qinzhi Wei, Jianhui Yuan, Zhiliang Zhu

**Affiliations:** 1 Department of Toxicology, Guangdong Provincial Key Laboratory of Tropical Disease Research, School of Public Health and Tropical Medicine, Southern Medical University, Guangzhou 528000, China; yungliu@126.com (Y.L.); zhangwj11@126.com (W.Z.); cnwei99@163.com (Q.W.); 2 Department of Occupational Health, Baoan Center for Disease Control and Prevention, Shenzhen 518100, China; doctoryangjp@163.com (J.Y.); gdhdl@163.com (Z.Z.); 3 Department of Environment and Health, South China Institute of Environmental Sciences, Ministry of Environmental Protection, Guangzhou 510655, China; pengxiaowu@scies.org; 4 Department of Toxicology, Shenzhen Center for Disease Control and Prevention, Shenzhen 518055, China; jianhui_yuan@hotmail.com

**Keywords:** MTBE, environmental pollution, inhalation exposure, toxicology, risk assessment

## Abstract

Methyl tertiary butyl ether (MTBE), a well known gasoline additive, is used in China nationwide to enhance the octane number of gasoline and reduce harmful exhaust emissions, yet  little is known regarding the potential health risk associated with occupational exposure to MTBE in petrol stations. In this study, 97 petrol station attendants (PSAs) in southern China were recruited for an assessment of the health risk associated with inhalation exposure to MTBE. The personal exposure levels of MTBE were analyzed by Head Space Solid Phase Microextraction GC/MS, and the demographic characteristics of the PSAs were investigated. Cancer and non-cancer risks were calculated with the methods recommended by the United States Environmental Protection Agency. The results showed that the exposure levels of MTBE in operating workers were much higher than among support staff (*p* < 0.01) and both were lower than 50 ppm (an occupational threshold limit value). The calculated cancer risks (CRs) at the investigated petrol stations was 0.170 to 0.240 per 10^6^ for operating workers, and 0.026 to 0.049 per 10^6^ for support staff, which are below the typical target range for risk management of 1 × 10^−6^ to 1 × 10^−4^; The hazard quotients (HQs) for all subjects were <1. In conclusion, our study indicates that the MTBE exposure of PSAs in southern China is in a low range which does not seem to be a significant health risk.

## 1. Introduction

In 1970s, the Clean Air Act (CAA) approved by the U.S. Congress banned the use of lead alkyls in gasoline, and methyl tertiary butyl ether (MTBE) was initially used as a substitute for lead alkyls to enhance octane number of gasoline and reduce carbon monoxide and hydrocarbon emissions [1,2,3]. Up to now, MTBE has been used commonly in many countries, including China. Epidemiological studies have shown that the blood concentrations of MTBE observed in exposed workers correlate positively with those of their working environment [4,5]. Oral, inhalation, and skin are the routes of human exposure to MTBE, with inhalation exposure representing the predominant occupational exposure source [1]. As observed in healthy human volunteers, MTBE is rapidly absorbed through the respiratory and digestive systems, efficiently distributed to various tissues through blood circulation, and metabolized within hours [2,6,7]. MTBE is metabolically activated by CYP2E1 and 2A6 (with equal importance) in rats [8], or mainly by CYP2A6 in humans [9,10,11,12], leading to the generation of tertiary butyl alcohol (TBA) as its major bioreactive metabolite.

Limited evidence in mammalian models indicates that MTBE might be genotoxic [13,14]. Chronic and high level exposure to MTBE (by inhalation or gavage) can cause various tumors in mice and rats [15,16,17,18]. However, there exists large uncertainty in the extrapolation of animal data to effects on humans [1,19,20], thus the evaluation of potential health effects based on actual exposure of humans to MTBE is essential. Indeed, early reports presented various complaints from residents in some MTBE-contaminated areas (New Jersey and Alaska in the U.S., where MTBE started to be added to fuel), including headache, cough, nausea, mucosal irritation, dizziness, and disorientation, *etc.*, suggesting potential health effects of MTBE [4,21]. However, a number of studies, both in field [22] and from laboratory work [23,24,25], did not show reliable links between MTBE exposure and these complaints. In short, differing conclusions among scientists regarding the potential health effects of MTBE still exist [1]; particularly, the health effects of MTBE exposure at occupational sites in developing countries such as China, have seldom been reported. 

The potential health risks to the population exposed to MTBE have received significant attention, but the conclusions remain unclear up to now. Health risk assessment of an ambient toxicant is a more trustworthy evaluation of the possible adverse effects caused by its exposure. Safety limits, expressed by parameters such as Acceptable Daily Intake (ADI), Minimal Risk Level (MRL) and Reference Dose/Concentration (RfD/RfC), are set up based on health risk assessments [26]. In 1991, the U.S. EPA established an inhalational RfC of 0.5 mg/m^3^ for MTBE [27]. In 1992, taking the effects of chronic exposures to MTBE into consideration, a revised RfC of 3 mg/m^3^, corresponding to an RfD of 0.42 mg/kg/day, was developed by the EPA and used as the benchmark for MTBE exposure. Below this concentration, MTBE is unlikely to cause an appreciable risk of non-cancer health effects in the general population [19,28,29]. Meanwhile, the average daily and lifetime average daily doses (ADD/LADD) of MTBE have also been estimated according to its air concentrations detected in 15 different occupational, commuting, or residential exposure categories in relevant areas of the U.S. [29]. With regard to MTBE, the American Conference of Governmental Industrial Hygienists (ACGIH) has established an occupational Threshold Limit Value (TLV) of 50 ppm. The present study was designed to conduct cancer and non-cancer risk assessments for inhalation exposure to MTBE in petrol station environments in southern China by using human health risk models [30,31].

## 2. Experimental Section

### 2.1. Study Population and Ethical Permission

The study population consisted of 97 petrol station attendants (PSAs) recruited during the period from August to October, 2013 from eight petrol stations located in Southern China, *i.e.*, petrol stations (P. station) A, B, C, D, E, F, G and H, corresponding to Beilong, Shihuagongmin, Huiguo, Tangwei, Baohongsheng, Baohua, Baoshi, and Shigongmin petrol stations, respectively. The selected subjects covered all the workers on duty during the days of our investigation. A spot field face to face investigation was performed on the employees of these petrol stations with self-made questionnaires. The individual characteristic data, including age, gender, body weight, type of job, working time per day, and length of exposure duration, *etc.*, were collected. The procedures for the recruitment and data collection were approved by the local ethical research committee (ethical code: 201158), and the purpose of the study was described in detail to each participant prior to obtaining written informed consent. 

### 2.2. Personal Exposure Sampling and Analysis of MTBE by GC-MS

Personal exposure sampling was conducted by using an organic vapor monitor (OVM; model 3520, 3M, St. Paul, MN, USA), a charcoal-based passive air sampler. The sampler was attached to the clothes of each participant (at the forechest) in the breathing zone (1.4 m). Each participant was sampled for three consecutive workdays (24 h) at his/her regular workplace. After sampling, the analytes were extracted from OVMs with carbon disulfide (Sigma-Aldrich, St. Louis, MO, USA) and then stored at −20 °C until being analyzed. Analysis of MTBE was performed with a Head Space Solid Phase Microextraction (HSSPME) GC/MS (model QP2010plus, Shimadzu, Kyoto, Japan), which is equipped with automatic AOC-20i + s sampler (Shimadzu) and a Rtx-Wax column (30 m × 0.32 mm × 0.25 μm). Helium was used as the carrier gas at a constant flow rate of 0.9 mL/min and in the splitless mode. An injection volume of 1 μL was run with an instant splitless time of 0.2 min. A direct analysis was performed after headspace extraction, with the inlet temperature set at 200 °C. The column temperature was initially maintained at 35 °C for 5 min, and then increased to 100 °C at a rate of 15 °C increase/min. The ion source temperature was set to 200 °C. The MS quadrupole temperature was set to 250 °C. The mass spectrometer was operated in the electron ionization mode (EI, 70 eV). Data acquisition was performed in full scan mode from *m*/*z* 20 to *m*/*z* 90 with a scan time of 0.5 s, at a mass accuracy of 0.1 atomic mass units. The acceleration voltage was turned on after a solvent delay of 1 min. The characteristic ions of MTBE at *m*/*z* 29, 41, 57, 73, with *m*/*z* 73 acting as a quantitative target ion for MTBE, and the other ions acting as a qualitative reference. 

### 2.3. Exposure, Cancer and Non-Cancer Risk Modeling

#### 2.3.1. Exposure Model

The following Equation (1) is used to calculate the Lifetime Average Daily Doses (LADD) (mg/kg/day) for inhalational exposure to air pollutants:

(1) $$LADD=\frac{C_{m}\times IR\times EL\times ED}{BW\times LT}$$

Estimation of the LADD using the above equation is based on the assumption that 100% of the inhaled pollutant is absorbed from the respiratory tract into the systemic circulation. As respiratory retention of MTBE has been estimated to be approximately 40% [7], calculation of MTBE-relevant LADD in this study had to be conducted according to the following Equation (2), obtained by modification of the original one:

(2) $$LADD=\frac{C_{m}\times IR\times EL\times ED}{BW\times LT}\times 40\%$$

where *C_m_* is the average concentration of personal exposure to MTBE in the petrol station environment (mg/m^3^), IR: inhalation rate (m^3^/h), EL: exposure length (h/day), ED: exposure duration (days), BW: body weight (kg), LT: life time (days). The sources of the various parameter values in the exposure model are listed in Table 1.

**Table 1:** Sources of parameter values in the exposure model.

Parameters	Values	Data Sources
C*_m_* (mg/m^3^)	Observed value	This study
IR (m^3^/h)	1.4	Value recommended by USEPA
EL (h/day)	8	This study
ED (days)	25 years × 365 days/year	Value recommended by USEPA
BW (kg)	Observed value	This study
LT (days)	70 years × 365 days/year	Value recommended by USEPA

#### 2.3.2. Cancer Risk Model

The Cancer Risk (CR) (assuming MTBE to be a potential genotoxic carcinogen) associated with inhalational exposure to MTBE was calculated according to Equation (3). CR is expressed as excess cancer risk after a lifetime inhalation of MTBE, which can be determined by normalizing the slope factor (SF) 1.8 × 10^−3^ (mg/kg/day)^−1^ for MTBE [32]. A more recent risk review has derived a SF of 2 × 10^−5^ (mg/kg/day)^−1^ for MTBE, which can be used for the calculation of CR (through multiplication of LADD by SF) [1]. The typical target range for risk management of 1 × 10^−6^ to 1 × 10^−4^ was adopted as the acceptable CR range [30]:

(3) Cancer Risk=LADD×Slope Factor

#### 2.3.3. Non-Cancer Risk Model

Non-cancer risk of inhalational exposure to MTBE was evaluated by the hazard quotient (HQ) method of risk characterization. The HQ was calculated by using the Equation (4). The RfD of 420 μg/kg/day (U.S. EPA benchmark) for inhalation exposure to MTBE, a level regarded as unlikely for the general population to be at appreciable non-cancer health risk, is adopted in Equation (4) [19,28,29]:

(4) $$Hazard\;Quotient=\frac{LADD}{RfD}$$

### 2.4. Statistical Analysis

SPSS 16.0 for Windows (SPSS Inc., an IBM Company, Chicago, IL, USA) was used for the statistical analysis of the obtained data. The independent-sample *t* test was used to compare the concentrations of inhalational MTBE between groups of operating workers and support staff. The data are expressed as mean ± S.D. and the standard for a statistical significance was *p* < 0.05.

## 3. Results and Discussion

### 3.1. Demographic Characteristics of Study Population

A total of 97 PSAs from eight petrol stations (petrol stations A through H) in southern China were recruited in this study. The individual characteristic data, including age, gender, body weight, type of job, working time per day, length of exposure duration, *etc.*, are listed in Table 2. The observed subjects from different petrol stations had similar demographic characteristics, as indicated by the mean age of subjects in various groups: 27.97 ± 6.72 (years) (ranging from 19 to 51 years); males (59) and females (38), comprising 60.82% and 39.18%, respectively; operating workers (61) and support staff (36) comprising 62.89% and 37.11%, respectively; the mean body weight being 56.77 ± 8.02 (kg); the length of exposure being 4.12 ± 3.83 (years); and their working time being consistently 8 h per day, 5 days per week. 

**Table 2:** Demographic characteristics of the study population *.

Characteristics	Individual Petrol Stations							
	A	B	C	D	E	F	G	H
Age (year)	29.25 ± 8.20	27.82 ± 7.61	28.82 ± 4.05	28.29 ± 8.70	27.00 ± 5.40	24.87 ± 4.72	26.25 ± 3.58	31.54 ± 6.13
Gender								
Male (%)	5(41.7)	5(45.5)	1(9.1)	8(57.1)	6(46.2)	8(53.3)	1(12.5)	4(30.8)
Female (%)	7(58.3)	6(54.5)	10(90.9)	6(42.9)	7(53.8)	7(46.7)	7(87.5)	9(69.2)
Body weight (kg)	55.13 ± 8.87	52.68 ± 4.12	61.09 ± 6.71	56.46 ± 6.79	54.53 ± 8.85	55.2 ± 5.29	62.65 ± 9.97	58.85 ± 9.33
Type of job								
Operating workers (%)	8(66.7)	6(54.5)	7(63.6)	9(64.3)	8(61.5)	10(66.7)	5(62.5)	8(61.5)
Support staff (%)	4(33.3)	5(45.5)	4(36.4)	5(35.7)	5(38.5)	5(33.3)	3(37.5)	5(38.5)
Working time (h/day)	8	8	8	8	8	8	8	8
Length of exposure (year)	2.63 ± 2.50	4.36 ± 4.12	6.32 ± 3.13	2.50 ± 2.01	4.27 ± 3.48	3.00 ± 2.80	4.88 ± 4.19	5.92 ± 3.77

* The number of subjects at P. stations A, B, C, D, E, F, G, and H were 12, 11, 11, 14, 13, 15, 8 and 13, respectively.

### 3.2. Concentrations of MTBE for Personal Exposure

**Figure 1:**
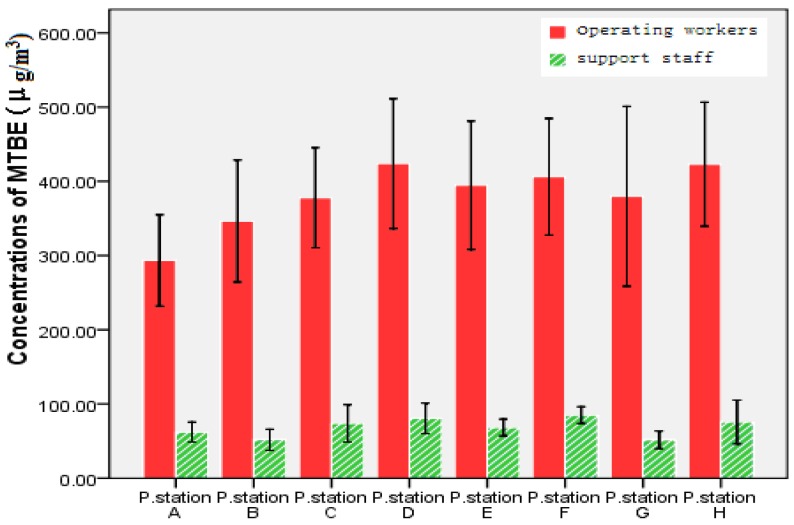
Average concentrations of personal exposure to MTBE in petrol station attendants.

**Figure 2:**
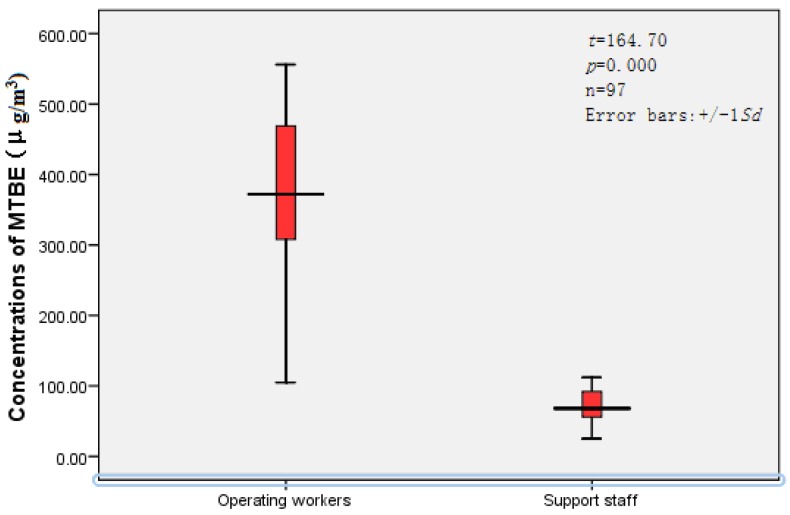
Average concentrations of personal exposure to MTBE in operating workers and support staff at the investigated petrol stations.

As shown in Figure 1, the concentrations of MTBE for personal inhalation were analyzed by HSSPME GC-MS. The mean MTBE exposure concentrations of the operating workers at petrol stations A through H were 293.46, 346.53, 377.89, 423.85, 394.67, 406.2 2, 379.89 and 423.03 μg/m^3^, respectively; while those of the support staff were 62.12, 51.65, 73.85, 80.70, 68.19, 85.00, 51.58 and 75.84 μg/m^3^, respectively, at the individual petrol stations. All of the observed values in each group were below the occupational TLV of 50 ppm (180,500 µg/m^3^). As shown in Figure 2, the mean values of MTBE exposure in the operating workers and support staff at all of the observed petrol stations were 388.38 μg/m^3^ and 71.61 μg/m^3^, respectively, and there is a statistically significant difference between them (*t* = 164.70, *p* < 0.01). 

It has been reported that the concentration of MTBE in the atmosphere of traffic, industrial, residential and commercial locations of the Pearl River Delta in Southern China ranged from 0 to 1.25 μg/m^3^, and the concentrations of MTBE in the urban air are relatively higher than in suburban areas [33]. These levels of environmental MTBE exposure seem to be close to those observed in other countries, e.g., 1 μg/m^3^ of MTBE in the air was detected in the public environment in the U.S. in 1997 [29]. However, the observed inhalational MTBE exposure levels at petrol stations in the present study are apparently lower than those observed in petrol service station attendants in the U.S., which were a few thousand of times higher than in the non-occupational environments. It is noticeable that in the recent 10 years the installation of air purification equipment at each petrol station has been enforced in southern China, a relatively developed region in China. This measure may have been effective in reducing the exposure levels of MTBE in petrol stations.

### 3.3. LADD (mg/kg/day) at C_m_ for Lifetime Inhalation of MTBE in Petrol Station Employees

The LADD (mg/kg/day) at C_m_ for inhalation exposure to air MTBE in the groups of operating workers and support staff was calculated by using Equation (2) and the results are shown in Table 3.

**Table 3:** Calculated LADD (mg/kg/day) at *C_m_* for MTBE exposure in operating workers and support staff (×10^−3^).

Type of Job	Individual Petrol Stations							
	A	B	C	D	E	F	G	H
Operating workers	8.52	10.52	9.90	12.01	11.58	11.77	9.70	11.50
Support staff	1.80	1.57	1.93	2.29	2.00	2.46	1.32	2.06

### 3.4. Cancer Risk Characterization with CR at LADD

Considering that the possibility for MTBE is a carcinogen has not been fully excluded [1], Equation (3) was used to calculate the CR for lifetime average daily doses by inhalation exposure to MTBE in petrol stations and the results are shown in Table 4. The calculated CR values in the investigated petrol stations ranged from 0.170 to 0.240 per 10^6^ for the operating workers and 0.026 to 0.049 per 10^6^ for the support staff. These values are all below the typical target range for risk management of 1 × 10^−6^ to 1 × 10^−4^ [30], therefore, the investigated workers and staff may not be at considerable cancer risk due to MTBE exposure in their occupational environment. The absence of significant cancer risk in MTBE-exposed population in the petrol stations in southern China may be related to the strengthening of environmental protection, pushed by the various levels of government administration in China in these years, particularly the special gasoline-air recycling equipment commonly used in petrol stations.

**Table 4:** Calculated CR per 10^6^ at LADD (mg/kg/day) for inhalation exposure to MTBE in petrol stations.

Type of Job	Individual Petrol Station							
	A	B	C	D	E	F	G	H
Operating workers	0.170 *	0.210	0.198	0.240	0.231	0.235	0.194	0.230
Support staff	0.036	0.0314	0.0386	0.046	0.040	0.049	0.026	0.041

* For example: 0.170 per million = 8.52 × 10^−3^ (mg/kg/day) × 2 × 10^−5^ (mg/kg/day)^−1^.

### 3.5. Non-Cancer Risk Characterization with HQ at LADD

As indicated in Table 5, the HQ values at LADD for non-cancer risk in the attendants of both job types in each petrol station were <1, *i.e.*, there was no considerable non-cancer risk for inhalational exposure to MTBE at the investigated petrol stations.

**Table 5:** Calculated HQ at LADD (mg/kg/day) for inhalation exposure to MTBE in petrol stations.

Type of Job	Individual Petrol Stations							
	A	B	C	D	E	F	G	H
Operating workers	0.020	0.025	0.024	0.029	0.028	0.028	0.023	0.027
Support staff	0.004	0.004	0.005	0.005	0.005	0.006	0.003	0.005

### 3.6. Limitations of This Study

The sample size of this study was relatively small, and therefore the representativeness of the sample is limited. Additionally, our study is an epidemiological cross-sectional survey, in which the sample collection was only performed during a limited time period, thus it can hardly shed light on health risks related to long-term exposure. It is encouraged that longitudinal surveys be carried out.

## 4. Conclusions

This study indicates that at the petrol stations in southern China the levels of inhalational exposure to MTBE of the petrol-refilling operating workers are several times higher than those of the support staff (*t* = 164.70, *p* = 0.000); Based on an analysis of the lifetime average daily dose of MTBE exposure in these petrol station attendants, little cancer risks and non-cancer risks exist (according to the typical target range for risk management of 1 × 10^−6^ to 1 × 10^−4^). Occupational exposure to MTBE in petrol stations in south China may not be a significant health hazard under the current administrative efforts.
